# Using the Blind Spot to Investigate Trans-Saccadic Perception

**DOI:** 10.3390/vision5030039

**Published:** 2021-08-26

**Authors:** Julie Royo, Fabrice Arcizet, Patrick Cavanagh, Pierre Pouget

**Affiliations:** 1 INSERM, CNRS, Institut du Cerveau, Sorbonne Université, 75013 Paris, France; julie.royo@icm-institute.org; 2 INSERM, CNRS, Institut de la Vision, Sorbonne Université, 75012 Paris, France; f.arcizet@gmail.com; 3 Psychology Department, Glendon College, CVR, York University, Toronto, ON M4N 3M6, Canada; patrick.cavanagh@gmail.com; 4 Department of Psychological and Brain Sciences, Dartmouth College, Hanover, NH 03755, USA

**Keywords:** trans-saccadic, saccade, vision, blind spot

## Abstract

We introduce a blind spot method to create image changes contingent on eye movements. One challenge of eye movement research is triggering display changes contingent on gaze. The eye-tracking system must capture the image of the eye, discover and track the pupil and corneal reflections to estimate the gaze position, and then transfer this data to the computer that updates the display. All of these steps introduce delays that are often difficult to predict. To avoid these issues, we describe a simple blind spot method to generate gaze contingent display manipulations without any eye-tracking system and/or display controls.

In this brief methodological report, we suggest a simple method to perform intra-saccadic display manipulation (such as double-step) without any eye-tracking system and/or display controls. The method addresses the timing challenges arising from the many steps required to update a display contingent on gaze. First, the online access to gaze position data is delayed. This end-to-end sample delay includes not only the time taken for a physical event to be registered, processed, and made available online by the eye-tracking system (e.g., capturing an image of the eye, fitting the pupil and corneal reflection, and extrapolating gaze position), but also the time needed to retrieve the data via Ethernet, USB, or analog ports. Second, as we need a reliable, and thus often a more conservative, criterion to decide whether a saccade has been initiated, the onset of the saccade detected online usually lags behind the onset of the saccade detected offline. Henceforth, this delay will be referred to as the saccade detection latency. Third, once we have detected a saccade in the data retrieved online, the stimulus has to be drawn to the graphics card’s back-buffer and the flip with the front-buffer has to be synchronized with the display’s vertical retrace [1]. This detect-to-flip latency is determined by the refresh rate of the monitor and depends on the time of detection within the refresh cycle. Fourth, there is the flip-to-display latency—that is, the time from the execution of the flip until the physical stimulus presentation on the screen. Whereas the transfer of the entire video signal takes up to one frame duration, the display’s reaction time can additionally increase the flip-to-display latency, as well as introduce temporal jitter. Because both gaze-contingent displays and saccade profiles can be subject to considerable variance, we increase the risk of the change occurring after rather than during the saccade. Failure to acknowledge or control these latencies can therefore lead to erroneous results and unwarranted conclusions.

We can avoid these complexities by exploiting our natural monocular blind spot to introduce gaze-contingent display changes. This new paradigm permits the study of trans-saccadic manipulations with little or no timing constraints. To do so, we simply use the natural blind spot observed in monocular vision as a self-generated saccade detector that produces an accurate intra-saccadic stimulus change. The blind spot corresponds to the optic disk on the retina, where blood vessels and ganglion-cell axons converge to form the optic nerve, which leads away from the eyeball to the brain. For this reason, the optic disk contains no photoreceptors (rods or cones), and thus no visual events can be registered within the blind spot [2,3,4]. The blind spot is located about 12–15° temporally and 1.5° below the horizontal and is roughly 7.5° high and 5.5° wide with some inter-individual variabilities. Although a stimulus on the display is not perceived when presented in the blind spot, it becomes detectable as soon as it exits the blind spot during the eye movement, avoiding the need to detect the saccade and update the display. 

As an example, we describe a version double step task using this paradigm, see Figure 1. The participants must initially fixate a central point and then move their gaze to a visible target. Shortly after the first saccade begins, the hidden second target is revealed as the blind spot has moved off of its location. After landing on the first target, participants then make a saccade to this newly visible point. This use of the blind spot offers the opportunity to explore diverse trans-saccadic manipulations in a simple setup, such as saccadic adaptation (using a flashed target outside the blind spot), saccadic inhibition (with masked stimuli within the blind spot) as well as visual remapping (with the remapped object or part of the object in the blind spot). Furthermore, although the blind spot encodes no visual information, one never perceives an odd dark or blank area there. Instead, one sees a complete scene of the world even when viewing monocularly [5].

## Figures and Tables

**Figure 1:**
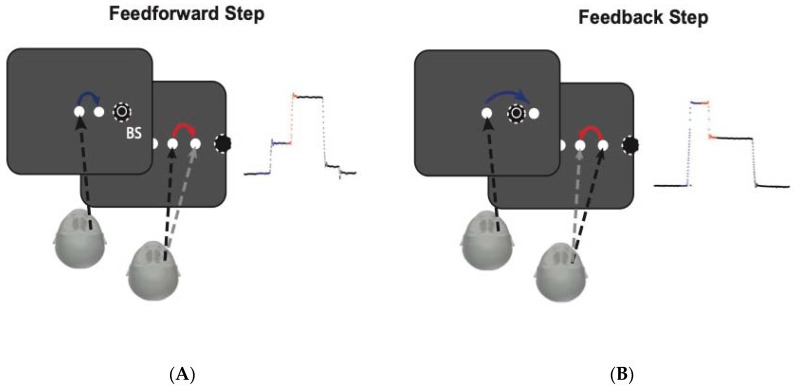
Example of double-step task using the blind spot to create the saccade contingent display changes: (**A**) The forward step task consists of a screen with three targets but the participants can only see two targets at first because the third target is located in the blind spot (BS). The participants initially fixate the central point and then move their gaze to the other visible point. Shortly after the first saccade begins, the hidden point to the right of the first target is revealed as the blind spot has moved away from its location. After landing on the first target, the participants then make a saccade to this newly visible point. The left panel shows the amplitude for eye position for each saccade (blue and red arrows). (**B**) The back step task is similar to the forward step with the difference that the hidden target in the blind spot is located between the two visible points. The participants initially fixate the central point and then move their gaze to the other visible point. Shortly after the first saccade begins, the hidden point to the right of the first target is revealed once the blind spot has moved away from its location. After landing on the first target, participants then make a saccade to this new point. The left panel shows the amplitude for eye position for each saccade (blue and red arrows).
